# Protein engineering strategies for improving the selective methylation of target CpG sites by a dCas9-directed cytosine methyltransferase in bacteria

**DOI:** 10.1371/journal.pone.0209408

**Published:** 2018-12-18

**Authors:** Tina Xiong, Dahlia Rohm, Rachael E. Workman, Lauren Roundtree, Carl D. Novina, Winston Timp, Marc Ostermeier

**Affiliations:** 1 Department of Chemical and Biomolecular Engineering, Johns Hopkins University, Baltimore, Maryland, United States of America; 2 Department of Biomedical Engineering, Johns Hopkins University, Baltimore, Maryland, United States of America; 3 Department of Cancer Immunology and Virology, Dana-Farber Cancer Institute, Boston, Massachusetts, United States of America; 4 Department of Medicine, Harvard Medical School, Boston, Massachusetts, United States of America; 5 Broad Institute of Harvard and MIT, Cambridge, Massachusetts, United States of America; Universität Stuttgart, GERMANY

## Abstract

Mammalian gene expression is a complex process regulated in part by CpG methylation. The ability to target methylation for *de novo* gene regulation could have therapeutic and research applications. We have previously developed a dCas9-MC/MN protein for targeting CpG methylation. dCas9-MC/MN is composed of an artificially split M.SssI methyltransferase (MC/MN), with the MC fragment fused to a nuclease-null CRISPR/Cas9 (dCas9). Guide RNAs directed dCas9-MC/MN to methylate target sites in *E*. *coli* and human cells but also caused some low-level off-target methylation. Here, in *E*. *coli*, we show that shortening the dCas9-MC linker increases methylation of CpG sites located at select distances from the dCas9 binding site. Although a shortened linker decreased methylation of other CpGs proximal to the target site, it did not reduce off-target methylation of more distant CpG sites. Instead, targeted mutagenesis of the methyltransferase’s DNA binding domain, designed to reduce DNA affinity, significantly and preferentially reduced methylation of such sites.

## Introduction

Cytosine methylation is important in facilitating many mammalian biological processes such as chromosomal stability, genomic imprinting, X-chromosome inactivation and gene expression [1–3]. Methylation has been implicated in embryonic development and cell differentiation [1, 3–5]. High levels of methylation in promoter regions often lead to transcriptional silencing, but many questions remain about the mechanisms by which DNA methylation alters gene expression. The ability to modulate methylation in a targeted way will facilitate the understanding of CpG methylation and its functional role in the context of specific cis-regulatory elements. As a result, protein fusions have been engineered that link 5-methyl cytosine methyltransferases to DNA binding domains (DBD) for the purpose of targeting methylation.

The typical strategy for targeting DNA methylation, as pioneered by Xu and Bestor [6], involves end-to-end fusion of a DNA binding motif and a cytosine DNA methyltransferase (typically human DNMT3a or bacterial enzymes). The DNA binding motif is designed to bind adjacent to the intended target region. The most commonly used DNA motif has been zinc fingers (ZF) [7]. With the advent of the CRISPR-Cas9 genome engineering tool, several groups, including ours, have recently reported on the targeting capabilities of fusions of catalytically deactivated Cas9 (dCas9) to DNMT3a or M.SssI [8–16]. A key feature of dCas9 as a DNA-binding motif is its ability to recognize a 20-nucleotide sequence defined by the single-stranded guide RNA (sgRNA) sequence that matches the protospacer and protospacer adjacent motif (PAM) on the DNA, thus avoiding the need for protein design required for ZF- and TALE-derived MTases. This affords greater flexibility in targeting different genomic regions; sgRNA synthesis can be done either commercially through oligo providers or via transcription from a synthesized DNA fragment/plasmid. Studies on dCas9-DNMT3a fusions demonstrate the potential of dCas9 to target DNA methylation in mammalian cells—guide RNAs can target methylation to different gene promoters; sgRNAs can be multiplexed to increase methylation throughout an entire genomic region, covering more of the promoter; methylation has demonstrated down regulation of target genes; and in some cases methylation has even been stably maintained after removal of dCas9-MT expression [8–11]. Together, these studies demonstrate proof of concept for gene regulation using Cas9 systems, and thus present the potential for transcriptional silencing of oncogenes. In addition, Shayevitch et al [16] used dCas9-DNMT3a-3l fusions to demonstrate that DNA methylation of exon-encoding regions is involved in the regulation of alternative splicing. Their study illustrates how dCas9-MT’s can be used to gain insight into the biological role of CpG methylation.

However, current tools demonstrate undesirable off-target activity [8–13, 15, 17, 18]. This off-target activity includes methylation at non-target CpG sites within a region near the target site (e.g. within a promoter) and at distal CpG sites in the genome. Two recent papers highlight the extent of the problem. Galonska et al. [15] recently provided the clearest evidence that dCas9-DNMT3a causes global increases in methylation by quantifying methylation in methylation-depleted but maintenance competent mouse ES cells, where a methylated genome is not an impediment to assessing off-target activity. The widespread off-target activity they observed was independent of guide RNA. Lin et al. [18] recently performed whole genome bisulfite sequencing for HEK293T cells expressing a dCas9-DNMT3a fusion and found over a thousand regions of off-target methylation, predominantly in promoters, 5’untranslated regions, and CpG islands. These sites only weakly correlated with predicted dCas9 off-target binding sites, suggesting that the problem primarily lies with the unregulated methyltransferase activity of the methyltransferase domain of the fusion. These two studies suggest that, although improving the targeting of the dCas9 domain is warranted, this will only partially address the problem. Approaches to improve selectivity for the target site over non-target sites are therefore needed. In addition, highly-specific programmable DNA methyltransferase would help basic science studies of the precise relationship between DNA methylation, its regulation and spread, and changes in gene expression. Improvements in targeting methylation have been made by substituting DNA targeting domains with higher DNA affinity or reducing methyltransferase activity [19–21]. However, methylation at non-target sites still occurs, most likely because the methyltransferase domain remains active even when the DBD is not bound at the target site. We reason such strategies might have better effects when used in combination with a methyltransferase whose methylation activity is dependent upon its assembly at the target site.

We have previously shown how naturally or artificially split methyltransferases is an attractive strategy for improving targeting ability [13, 22, 23]. Most recently, we have demonstrated the targeting abilities of dCas9 fused to a M.SssI split methyltransferase (sMTase) [13]–a split M.SssI that had been used previously in the context of zinc finger instead of dCas9 [19, 22]. In our dCas9-sMTase, dCas9 is fused to the N-terminus of the C-terminal fragment encoding M.SssI [273–386] (MC). We chose to leave the N-terminal fragment encoding M.SssI [1–272] (MN) untethered to a DBD, because this reduced the number of PAMs that needed to be located at an appropriate distance from targeted CpG sites. We hypothesized that when the dCas9 domain is not bound to DNA, the two M.SssI fragments would lack sufficient stability or affinity for each other to efficiently methylate DNA. When the dCas9 is bound to the sgRNA-determined DNA site, localization of the MC domain would increase its stability via interactions with the CpG site or induce its folding in cooperation with MN.

In previous work [13], we first characterized our constructs in *E*. *coli*, because these cells lack native mechanisms for CpG methylation. These experiments showed how methylation efficiency at the target site varies as a function of its distance from the PAM site and differs for the cis and trans strands (the cis strand is defined as the strand containing the PAM site). The resulting model for methylation efficiency as a function of the gap between the PAM and the CpG site was reasonably predictive of the targeted methylation efficiency in genomic DNA in HEK293T cells. Guide RNA-directed methylation efficiency at target sites was as high as ~40% in *E*. *coli* and ~70% in HEK293T cells. Methylation at non-target CpG sites was generally low (~1% in *E*. *coli* and 0–3% above background in HEK2393T). However, we observed methylation frequencies as high as 13% at select non-target sites within a few hundred nucleotides of the target site on plasmids in *E*. *coli*. Similarly, in HEK293T we had one instance of methylation levels of 10–25% at a group of four non-target CpGs ~200 nucleotides away from the target site. Our evidence suggested that this type of non-target methylation primarily occurs with dCas9 bound at its target protospacer site, as opposed to dCas9 bound at an unintended site or dCas9 not bound to DNA at all. Our evidence further suggested that non-target sites with significant methylation are sites that are within reach of dCas9-sTMase bound at its intended site due to DNA topology in the cells, such as plasmid supercoiling in *E*. *coli*. dCas9-MC/MN may also cause non-target methylation due to inherent issues with dCas9 specificity, but we did not observe such off-target activity. This is likely due to the low probability of having a promiscuous dCas9 binding site given that our methylation analysis was limited to the ~4 kb of an *E*. *coli* plasmid and the several hundred bp in the SALL2 and HBG promoters in HEK293T.

Here, we chose to improve methylation specificity of dCas9-MC/MN by reducing off-target methylation in the vicinity of the target site. We chose to test our hypothesis for improving specificity in *E*. *coli*, since its lack of native CpG methylation allows for unambiguous attribution of the enzyme methylating the CpG site (i.e. endogenous methylation enzymes in human cells complicate such analysis). By comparisons to our previous work [13], we show that methylation of the target site can be increased by shortening the linker, and that bias for methylation at the target site can be improved by mutations designed to reduce the interaction of the MC fragment with the DNA. Both of these strategies reduced the number of PAM-CpG distances at which effective targeting occurred in *E*.*coli*. We hypothesize that these linker and mutational changes will cause similar effects for dCas9-MC/MN targeted methylation in human cells based on our previous comparisons of dCas9-MC/MN’s performance in *E*. *coli* and that in human cells. However, this hypothesis needs to be tested in human cells.

## Results and discussion

### The effect of linker length between dCas9 and MC

**Fig 1:**
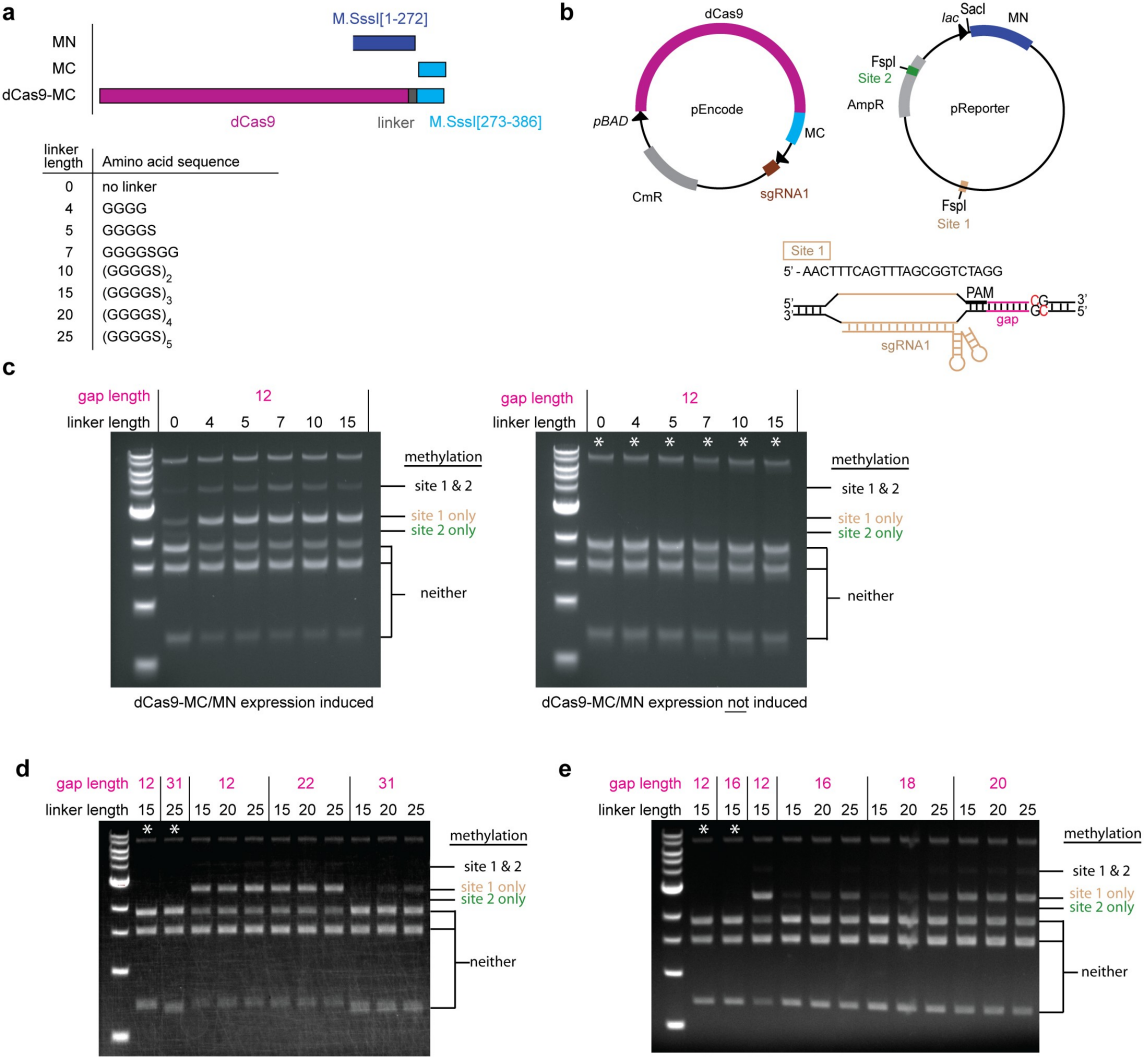
Effect of linker length on methylation targeting in ER2267 *E*. *coli* cells. (a) Schematic of protein constructs and linkers used in *E*. *coli* studies. (b) Methylation was tested in a two plasmid ER2267 system with inducible dCas9-MC/MN genes and constitutively expressed sgRNA1. Each of site 1 and site 2 contains a CpG site imbedded in an FspI site to allow screening of methylation by FspI endonuclease. Plasmid DNA isolated from cells containing this plasmid pair and expressing sgRNA1 were subjected to in vitro restriction enzyme protection assay to test (c) the effect of shortening the linker length on methylation at a fixed gap distance (d) the effect of lengthening the linker on methylation at longer gap distances, and (e) the effect of lengthening the linker on methylation at gap distances for which the dCas9-MC/MN enzyme with the 15 aa linker is ineffective at methylation. sgRNA1 is designed to target methylation to site 1. The sizes of bands corresponding to methylation at the indicated sites are specified to the right of the gel images. The asterisk indicates that dCas9-MC/MN expression was not induced. All plasmid DNA was digested with SacI (to linearize the plasmid) and FspI (to test for methylation at sites 1 and 2). Plasmid pEncode lacks FspI restriction enzyme site and is the band at the top of the gel.

In our previous work [13], we used a 15-amino acid linker between dCas9 and MC. We hypothesized that shortening the linker might better restrict methylation to the target CpG site in the sequence immediately downstream of the protospacer. We tested linkers ranging in length between 0 and 25 amino acids. As in our previous work, we assayed methylation activity in *E*. *coli* using a two-plasmid system expressing dCas9-MC and MN under inducible promoters and the sgRNA under a constitutive promoter (Fig 1a and 1b). The pReporter plasmid contains two FspI sites that have CpGs embedded within them, which upon methylation are blocked from FspI endonuclease digestion. Guide RNA sgRNA1 targets one of the FspI sites (site 1) containing a CpG site 12 nucleotides away from the PAM (Fig 1b). The FspI site within the *amp*^*R*^ gene (site 2) is naturally occurring and the surrounding DNA is not a match for sgRNA1.

**Fig 2:**
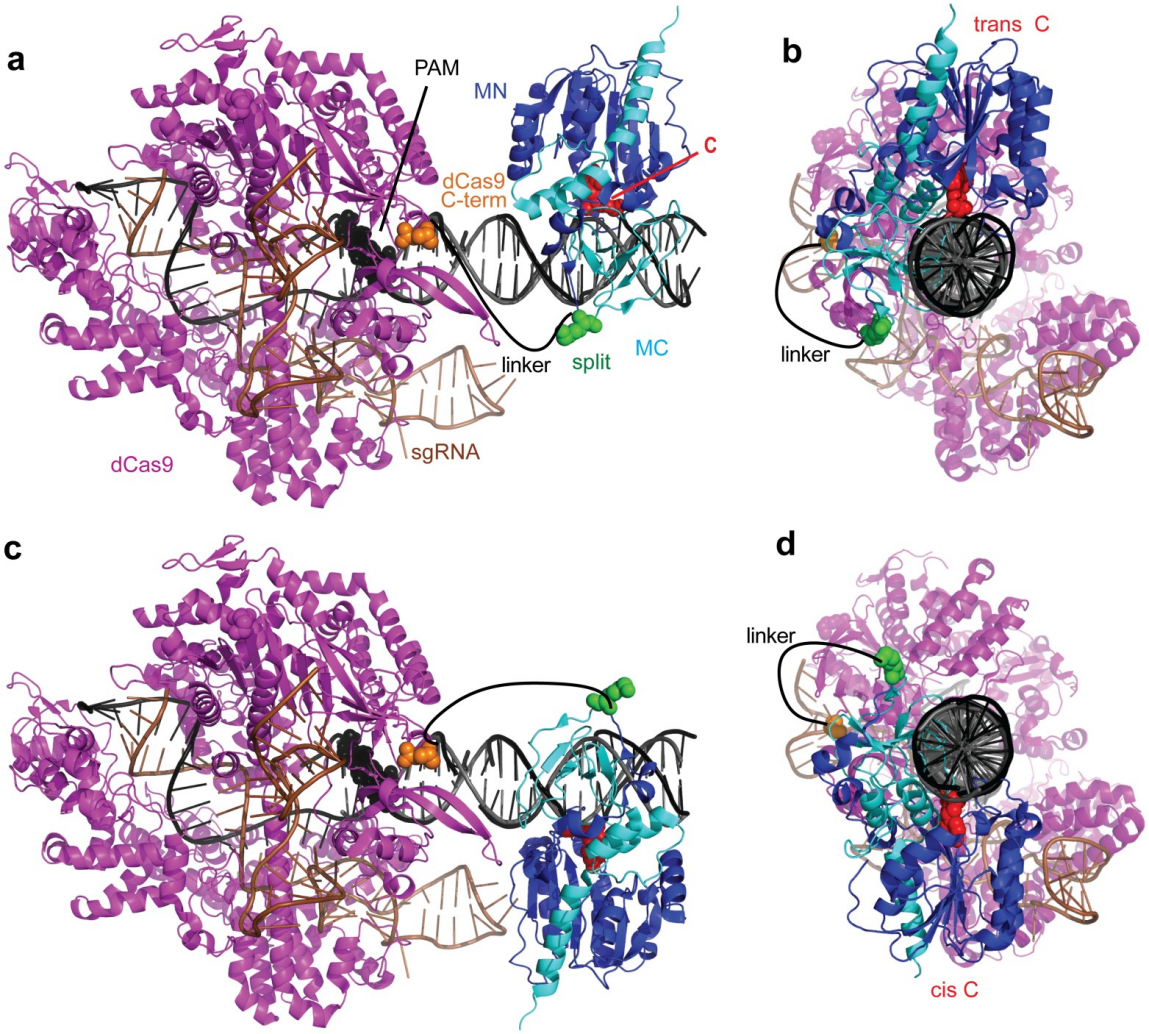
Structural models of dCas9 and M.SssI bound to the same DNA. The model shows a gap length of 11 nucleotides between the PAM and the target CpG site. Models were built by superimposing the crystal structure of DNA-bound *S*. *pyogenes* dCas9 (4UN3) [24] and the M.SssI homolog M.HhaI (2HR1) [25] onto the same molecule of B-DNA. (a) Front view and (b) side view of M.HhaI bound on the trans strand 11 base pairs from the PAM. (c) Front view and (d) side view of M.HhaI bound on the cis strand 11 base pairs from the PAM. A hand-drawn line represents the linker between the C-terminus of dCas9 and the N-terminus of MC. Figure adapted from Xiong et al. [13].

We isolated plasmids from cultures of ER2267 *E*. *coli* cells co-expressing dCas9-MC/MN and sgRNA1, and methylation was assessed by restriction protection assay using FspI endonuclease (Fig 1c–1e). In this qualitative screen, we did not observe an obvious difference in the pattern of protection at a gap length of 12 nucleotides when the linker was shortened to 10, 7, 5, or 4 amino acids (Fig 1c) or when the linker was lengthened to 20 or 25 amino acids and gap lengths of 12 and 22 nucleotides were used (Fig 1d). Lengthening the linker from 15 to 20 and 25 amino acids appeared to offer progressively more protection at site 1 at a gap length of 31 nucleotides, a length at which dCas9-MC/MN with a 15-amino acid linker is ineffective at methylation [13] (Fig 1d). This observation suggested the obvious possibility that a longer linker length might be able to reach more distant sites. At gap lengths of 16 and 18 nucleotides, lengths at which dCas9-MC/MN with a 15-amino acid linker is ineffective at methylation [13], lengthening the linker from 15 to 20 and 25 amino acids appeared to offer progressively more protection at site 1. This observation suggested that longer linker might be able to reach CpG sites at gap lengths that our structural model (Fig 2) suggested would require the linker to wrap around the DNA [13]. Based on these preliminary screens, we chose linker lengths of 4, 15, and 25 amino to quantitatively study the effect of linker length on CpG methylation by dCas9-MC/MN.

**Fig 3:**
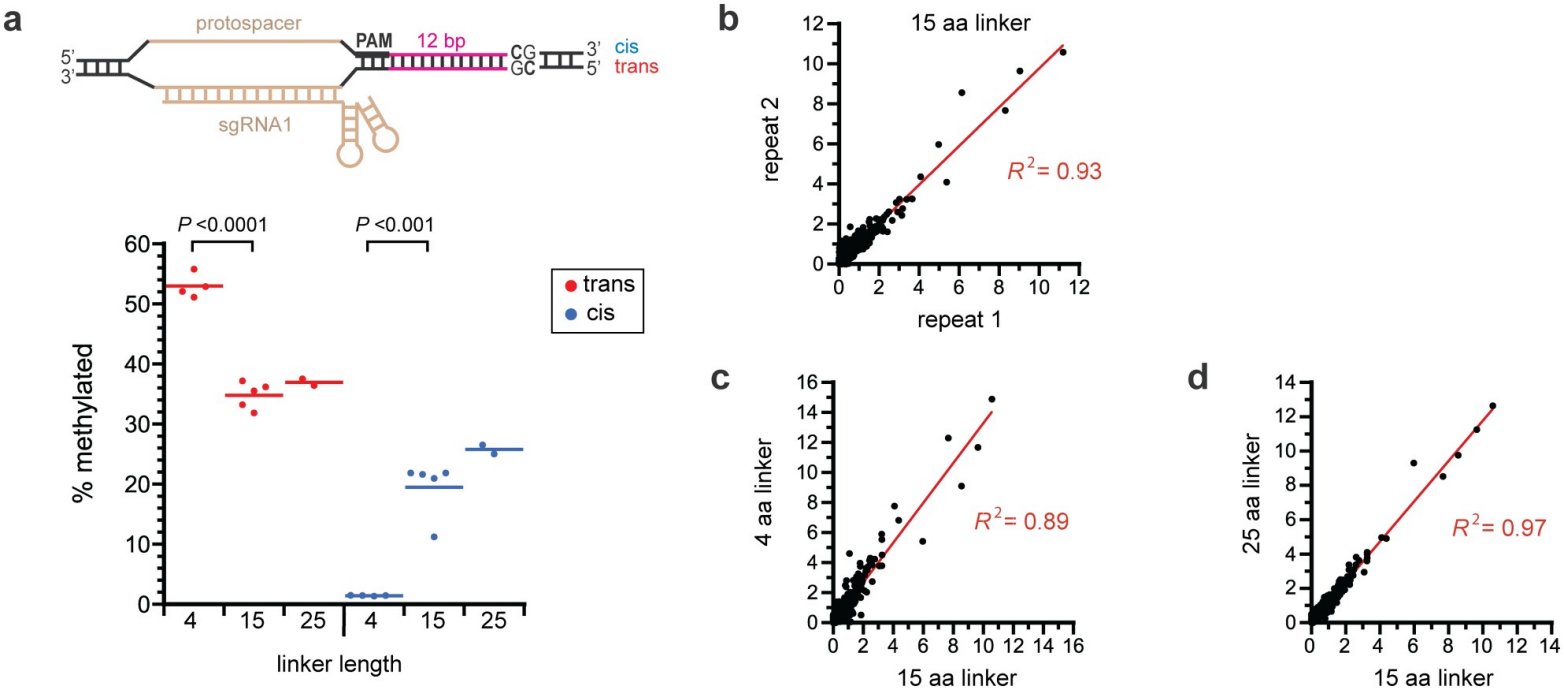
Effect of linker length on methylation at the target site and at non-target sites. (a) Frequency of methylation caused by dCas9-MC/MN with different linkers at the target CpG site at a gap length of 12 nucleotides from the PAM site. The *P* values are from Student’s *t*-tests (*n* = 4 for the 4-amino acid linker and *n* = 5 for the 15-amino acid linker). Four of the five measurements for the 15-amino acid linker are from [13] and one is new. (b) Reproducibility of the frequency of methylation at 482 C’s in 241 non-target CpG sites on plasmid pReporter. Data is for the 15-amino acid linker (repeat 1 is from our previous study [13]; repeat 2 is new). (c) Correlation of off-target methylation levels when using a 4-amino acid linker and 15-amino acid linker. (d) Correlation of off-target methylation levels when using a 15-amino acid linker and 25-amino acid linker. Frequencies were determined by high-throughput bisulfite sequencing.

We previously use bisulfite sequencing to quantify methylation at the target CpG site on a plasmid in *E*. *coli* when the 15-amino acid linker was used [13]. Here, we do the same for 4- and 25-amino acid linkers and compare this to our previous data to see how linker length affects methylation frequency at the target CpG site (Fig 3a). We include data from a new repeat of our experiments with the 15-amino acid linker to ensure a comparison to our previous experiments is justified (Fig 3a and 3b). Methylation of each cytosine in a CpG site requires MN/MC to bind in different orientations (Fig 2), so we determined the frequency of methylation on both strands of the target site. To unambiguously define the two strands in relation to the PAM site, we designate “cis” as the PAM/protospacer-containing DNA strand and “trans” as the sgRNA-complementary. For a 12-nucleotide gap between the PAM and the CpG site, shortening the linker from 15 to 4 amino acids increased methylation 52% on the trans strand but decreased methylation by 93% on the cis strand (Fig 3a).

Our bisulfite data also allowed us to measure methylation levels at the 241 non-target CpG sites on pReporter. The levels of off-target methylation were consistent between replicate experiments, including which off-target sites received the highest levels of methylation (Fig 3b). We found that neither shortening nor lengthening the linker appreciably changed the level of methylation of non-target sites or altered which off-target sites received the highest levels of methylation (Fig 3c and 3d). The median level of methylation at non-target sites for all 3 linkers was below 1% (0.60 ±0.10 for the 4-amino acid linker, 0.62 ±0.20 for the 15-amino acid linker and 0.59 for the 25-amino acid linker).

**Fig 4:**
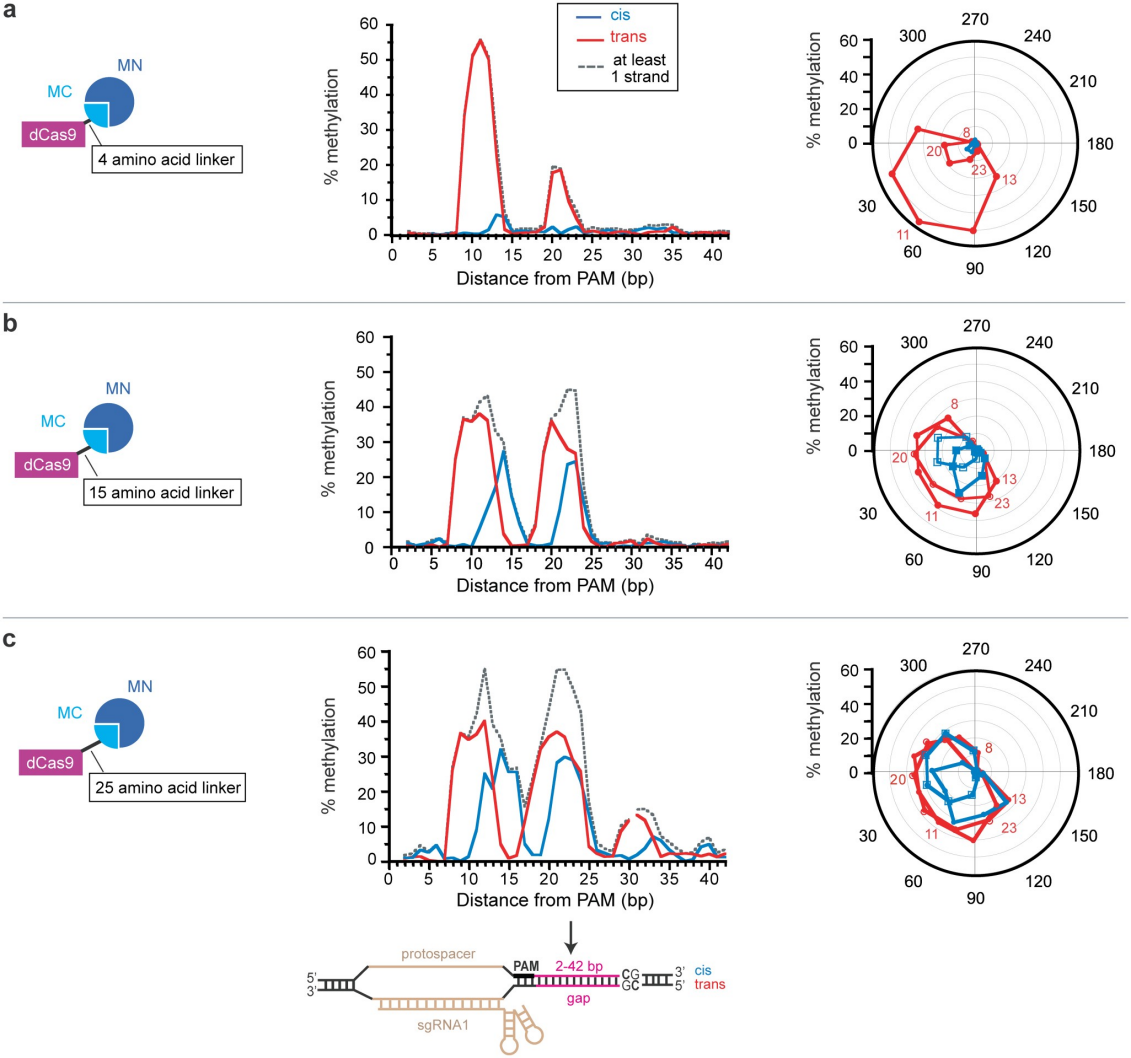
Effect of linker length on methylation in the target region as a function of gap length. Frequency of methylation for cis and trans strand are presented as a function of distance from the PAM (center) and the angle between the dCas9 C-terminus and the MC N-terminus (right) in the structural model for dCas9-MC/MN for a (a) 4-amino acid linker, (b) 15-amino acid linker, or (c) 25-amino acid linker. The angle is defined as that formed between the C-terminus of Cas9 and N-terminus of MC when looking down the DNA axis from the CpG site towards the PAM (i.e. the angle formed in the plane of the page in the view of Fig 2b and 2d). Full amino acid sequences of linkers can be found in Fig 1a. The dotted line indicates the fraction of the target sites that are methylated on at least one strand assuming that cis and trans strand methylation is independent. For c, methylation frequency data was not obtained for the trans strand for the gap lengths of 31 and 37 bp. Data in (b) is from Xiong et al. [13].

To examine how linker length affected the ability of dCas9-MC/MN to methylate CpG sites at different “gap lengths” (i.e. different distance in nucleotides between the PAM and the CpG site), we determined the frequency of methylation on the cis and trans strand cytosines in target sites located at a gap length of 2 to 42 nucleotides from the PAM site. We used our previously described series of 40 pReporter plasmids in which the target CpG site was located at 40 different distances from the PAM site [13]. We co-cultured the 40 different cells carrying these pReporter plasmids (and pEncode) and subjected these pReporter plasmids to high-throughput bisulfite sequencing. In our previous study [13] with the 15-amino acid linker, methylation required a gap length of at least 8 base pairs and no more than 26 base pairs distance and oscillates as a function of distance with a periodicity of 11 base pairs (Fig 4b). This finding was in accordance with structural models, as methylation was favored when the fusion sites of MC to dCas9 are located on the same side of the double helix, presumably because the linker does not have to wrap around the DNA [13]. Here, by comparison with our previous study [13] we find that shortening the linker between the dCas9 and MC from 15 to 4 amino acids restricted the set of distances between the PAM and the CpG that are compatible with methylation (Fig 4a). As expected, shortening the linker decreased methylation at longer gap lengths. Additionally, shortening the linker almost completely eliminated cis-strand methylation. High levels of trans-strand methylation occurred only in a narrow range of gap lengths of 10 to 12 nucleotides. Methylation levels in this optimal gap length range were higher for the 4-amino acid linker (52.4 ± 2.9%) than for the 15-amino acid linker (36.7±1.2) (*P* < 0.0001, Student’s *t*-test). We speculate that shortening the linker may increase DNA methylation at the most favorable distance for methylation by decreasing conformational entropy.

We hypothesize that a longer linker between the dCas9 and the methyltransferase domain might be used to target a wider set of CpG sites in a single region. By comparison with our previous study using the 15-amino acid linker [13], we find that lengthening the linker to 25 amino acids increased the number of distances between the CpG site and the protospacer that are compatible with methylation (Fig 4c). The longer linker increased cis-strand methylation (*P*<0.0001 by paired Wilcoxon rank-sum test) and resulted in methylation at the target CpG site as high as 55% at the optimal gap length. Additionally, the use of a long linker caused methylation at gap distances that were otherwise too far to reach when the 15-amino acid linker was used.

Although shortening the linker increased methylation efficiency on the trans strand at CpG sites 10–12 nucleotides away from the PAM, this came at the expense of methylation efficiency at all other gap lengths. Furthermore, shortening the linker from 15 to 4 amino acids did not decrease methylation levels at most non-target sites (Fig 3c) but reduced the ability to methylate CpG sites that were very close to the target site (Fig 4). Perhaps, more distant non-target CpG sites that are brought near the target site due to DNA topology can adopt a wide array of orientations relative to the MC domain (thus allowing methylation to occur) or the C-terminus of the dCas9 may serve as a linker that enable MC access to these non-target sites. In contrast, CpG sites proximal to the target site that are at non-optimal gap lengths from the PAM cannot be reached due to their proximity to the dCas9 binding site and the persistence length of DNA.

### Mutations in the methyltransferase domain that improve methylation targeting in *E*. *coli*.

**Fig 5:**
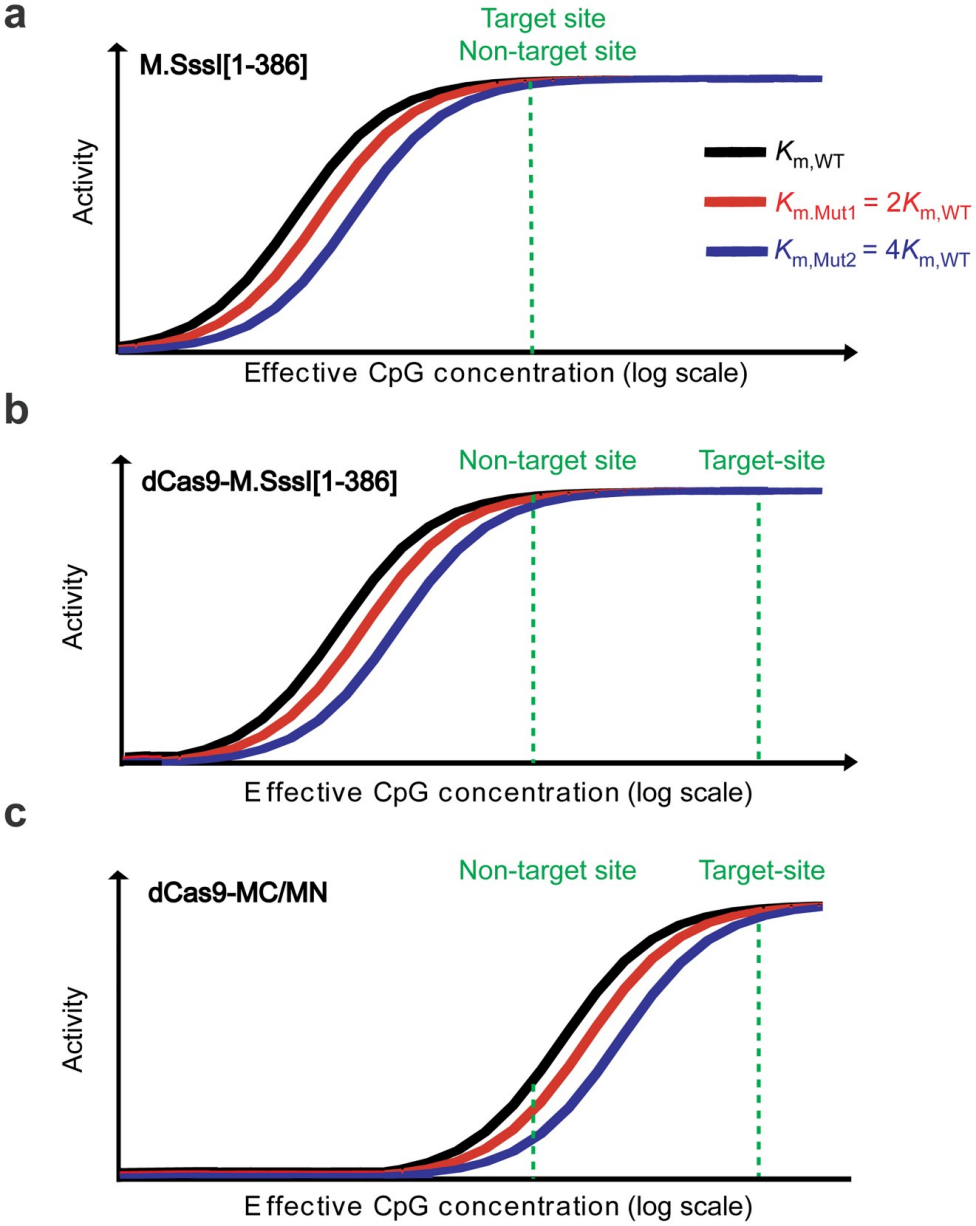
Model for how affinity-weakening mutations improve methylation specificity. Schematic representation of the effect on methylation activity of mutations that reduced a methyltransferase’s binding affinity to DNA for (a) M.SssI (b) dCas9-M.SssI, and (**c**) dCas9-MC/MN. Methylation activity of the methyltransferase (black curve) and the associated variants with reduced affinity for the DNA (red curve and blue curve) as a function of the effective CpG concentration as calculated using the kinetic mechanism of homologue methyltransferase M.HhaI [27]. Effective concentration is plotted in log scale. The three curves differ in the value of *K*_m_ for DNA as indicated. The green dashed lines indicate hypothetical effective concentrations of target and non-target sites, as indicated. These lines reflect the fact that dCas9 directing dCas9-M.SssI and dCas9-MC/MN to the vicinity of the target CpG site will increase the effective concentration of the target CpG site.

The most effective mutation to improve the ratio of methylation at the target CpG site to methylation at the non-target site is one that preferentially reduces the catalytic rate at the non-target site. Based on previous studies [19, 21], we reasoned that mutations that reduce the methyltransferase domain’s affinity for DNA would reduce the overall rate of methylation, but with more pronounced effects at non-target sites thereby improving the specificity of our engineered enzyme. This improvement in specificity arises from a combination of the increase in the *K*_m_ for DNA and the increase in the effective concentration of target CpG sites when the methyltransferase is tethered to a DNA binding domain bound at an appropriate distance from the target CpG site. In the absence of tethering (Fig 5a), target and non-target sites are kinetically indistinguishable and a reduction of *K*_m_ affects both equally. When the methyltransferase is tethered, the effective concentration of a target CpG sites increases, provided it is at the appropriate distance away from the dCas9 binding site (Fig 5b and 5c). Such a method in theory should be equally effective for dCas9 fused to full-length M.SssI or to MC—what matters is whether the mutation increases the *K*_m_ enough to observe the effect. Split methyltransferases might be poised to exhibit improvement due to their being catalytically compromised by the act of splitting the enzyme (Fig 5b vs. Fig 5c). Note that in this model, the *K*_m_-increasing mutation does not increase the inherent specificity of the enzyme. The mutation is just a means to move the enzyme into a regime where in the context of the cell (with a given concentration of DNA and enzyme) the preferential methylation of target site over non-target site can be best achieved. This model explains why the Q147L mutation in dCas9-MQ1 (an end-to-end fusion of dCas9 and M.SssI) helps its targeting [20], as the mutation is known to reduce catalytic activity through disrupting interactions with DNA [26]. [20]

**Fig 6:**
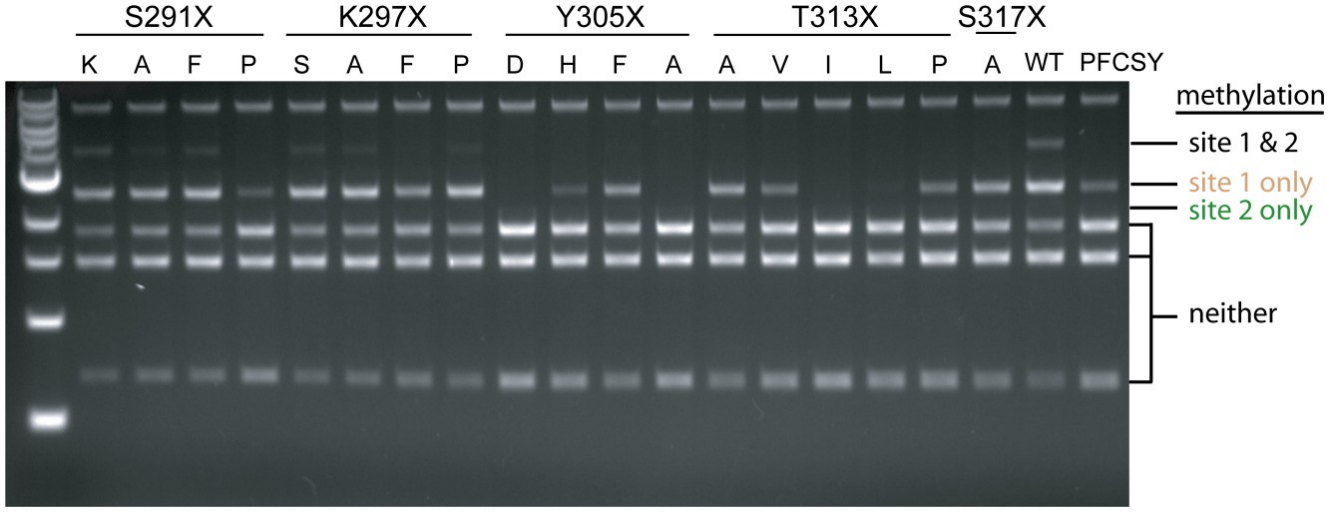
Methylation of dCas9-MC/MN and associated variants as assessed by FspI restriction protection assay. sgRNA1 was used to target methylation to site 1. Methylation was assayed from plasmid DNA extracted from ER2267 cells expressing the dCas9-MC/MN (WT) or the indicated dCas9-MC/MN variant. The first row indicates the position mutated, and the second row indicates the mutation made. The PFCSY set of mutations is in the final lane.

To improve the ratio of methylation at the target site to that at non-target sites, we chose non-conservative mutations at positions in the MC domain based on a model of M.SssI/DNA complex, sequence alignments of different C5-MTases, and previous mutational analysis of M.SssI [26, 28, 29]. We chose to introduce mutations at five positions that interact with DNA: S291, K297, Y305, T313 and S317. In addition, we introduced the K297P/N299C/E301Y triple mutation (referred to as PFCSY in the original paper) identified in our previous study of mutations that improved the specificity of a split M.SssI enzyme fused to zinc finger proteins [19]. We used the FspI restriction digest protection assay on pReporter [13] as a screen to identify mutations that still had relatively high levels of methylation at the target site but no detectable methylation at a non-target site. This assay suggested that all mutations reduced methylation at the non-targeted FspI site, some mutations eliminated methylation altogether, and some mutations might preferentially reduce methylation at the non-target FspI site (Fig 6).

**Fig 7:**
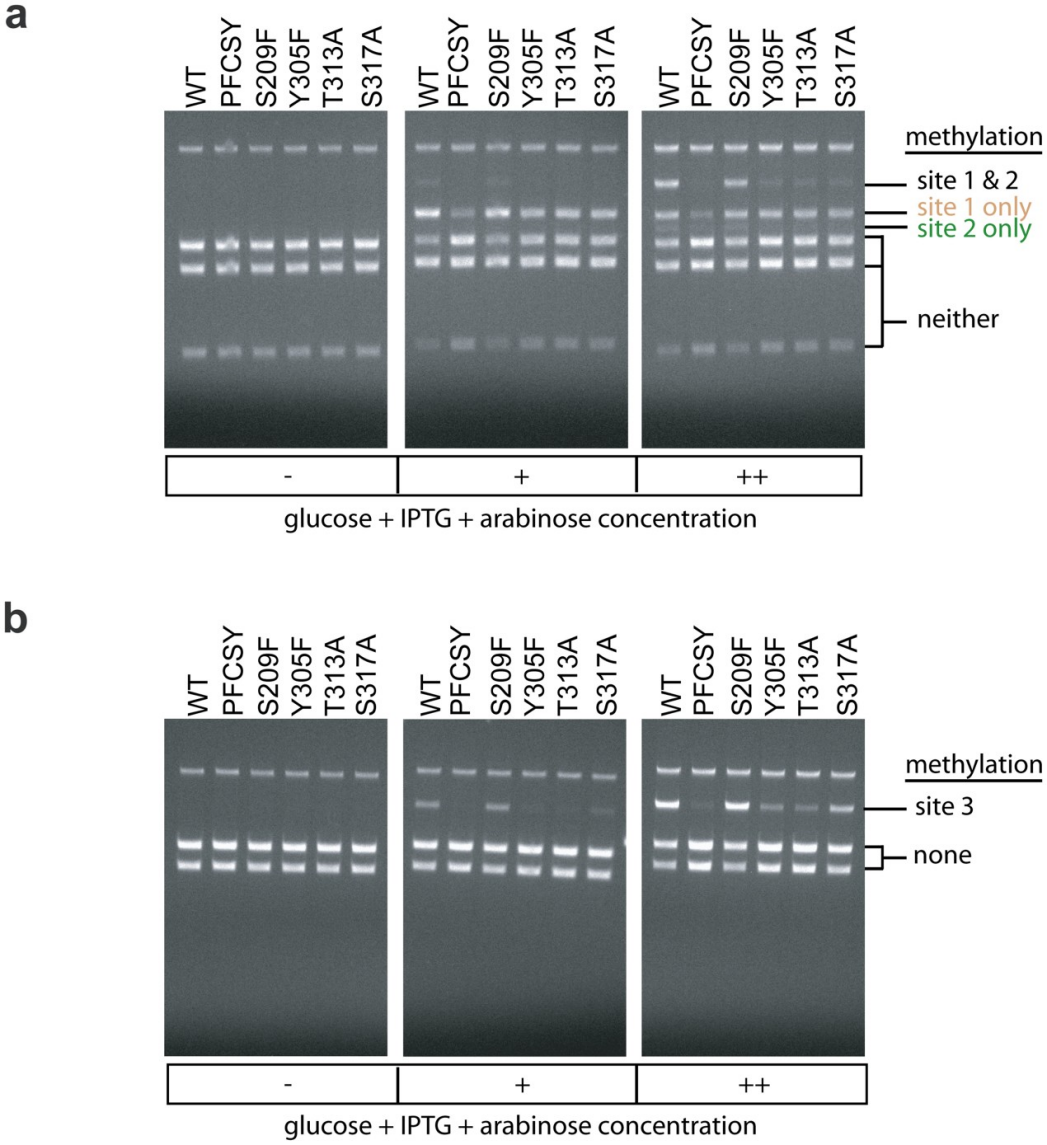
Effect of expression level of dCas9-MC/MN and mutations in MC on methylation activity and specificity. Methylation was assayed by (a) FspI restriction protection assay on sites 1 and 2 and (b) SnaBI restriction protection assay on site 3. sgRNA1 targeted methylation to site 1. Methylation was assayed using isolated plasmid DNA from cells grown in LB media containing (-) 0.2% glucose, (+) 0.2% glucose + 1mM IPTG + 0.0167 arabinose, (++) 0.02% glucose + 1mM IPTG+ 0.0167% arabinose. These supplemented compounds affect the expression of the two fragments of the methyltransferase as explain in Fig 1b and the main text. The symbols (-, +, ++) also qualitatively indicate the expected level of dCas9-MC/MN expression.

Based on this preliminary screen, we chose five mutations for further screening: S209F, Y305F, T313A, S317A, and PFCSY, as these appeared to be best at retaining digestion protection at site 1 and reducing protection at site 2. For these additional screens, we decreased the amount of glucose in cultures, as we observed that this increased the level of methylation observed for induced cultures allowed to grow overnight. This methylation level increase is presumably due to glucose’s known catabolite repression of *lac*- and *pBAD*-derived promoters. The decrease in glucose concentration allowed us to better assess the effect of the mutations on methylation of specific non-target sites by restriction enzyme protection assays (Fig 7a). For a further screen on these five mutants, we modified the nucleotides flanking an off-target CpG site on pReporter observed to have high methylation. This modification created a SnaBI site, which we refer to as site 3. SnaBI endonuclease is blocked by methylation at its internal CpG site. We assessed methylation of this non-target site by SnaBI protection assay for select mutants (Fig 7b). In these two additional restriction enzyme protection assay screens, Y305F and T313A appeared to perform the best. They appeared to exhibit a combination of comparable levels of protection from digestion at site 1 (the target site) and reduced protection at sites 2 and 3 (Fig 7). Based on these preliminary, qualitative screens, we selected Y305F and T313A for more careful quantitative analysis of their effect on methylation.

**Fig 8:**
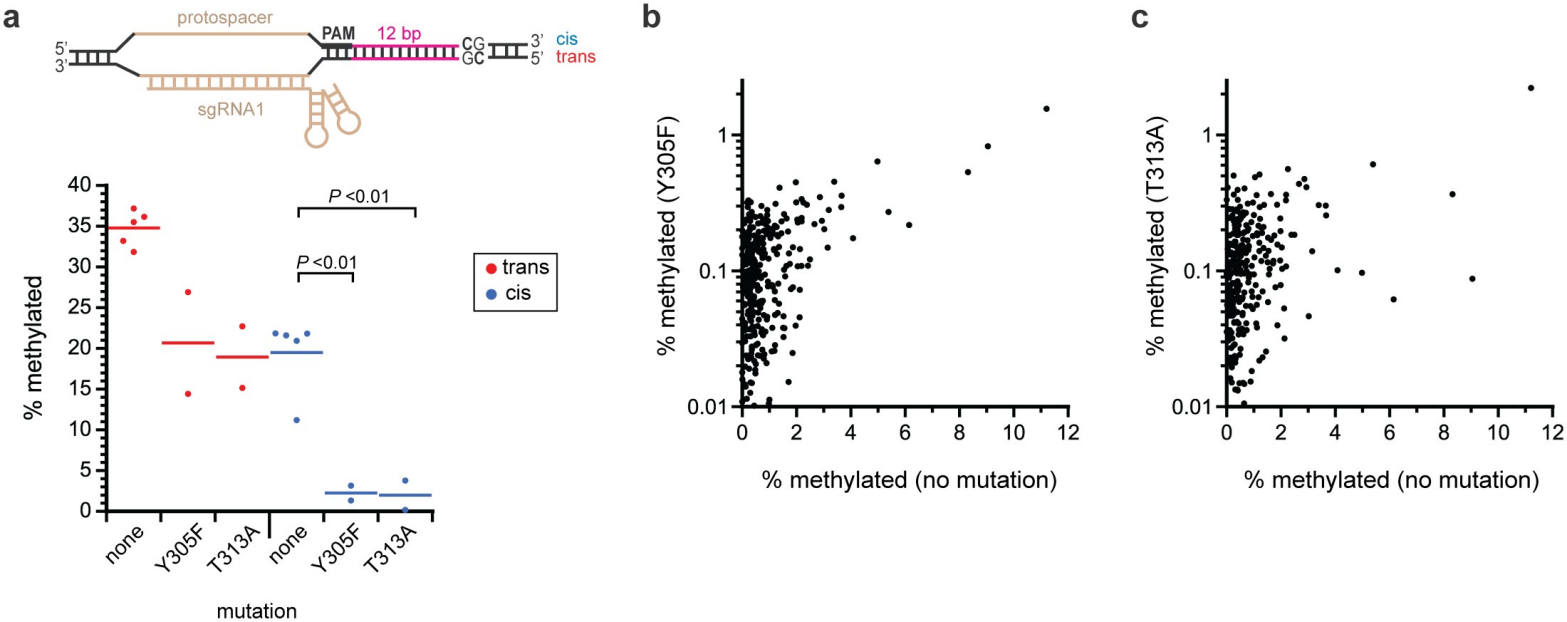
Effect of select mutations on methylation at the target site and at non-target sites. (a) frequency of methylation the target CpG site at a gap length of 12 nucleotides from the PAM site. The *P* values are from Student’s *t*-tests (*n* = 5 for the no mutation and *n* = 2 for Y305F and T313A). Four of the five measurements for no mutation are from [13] and one is new. Comparison of the effect of the (b) Y305F and (c) T313A mutations on the frequency of off-target methylation at 482 C’s in 241 non-target CpG sites on plasmid pReporter. Frequencies were determined by high-throughput bisulfite sequencing.

We next subjected Y305F and T313A to more in-depth characterization by high throughput bisulfite sequencing of the entire pReporter plasmid. We placed the target site at a gap distance of 12 nucleotides from the PAM in these experiments. We quantified the frequency of methylation on each strand at the target CpG site and the 241 other non-target CpG sites in the plasmid. We then compare this to our previous study [13] and new data with constructs lacking a mutation. Both mutations decreased methylation at the target site, but the decrease was much more pronounced for methylation of the cis strand (Fig 8a). The mutations reduced target site trans strand methylation only about 50% (Fig 8a), but reduced off-target methylation at the 241 non-target sites many-fold, as desired (Fig 8b and 8c). For each mutant, only one of the 482 C’s in off-target CpG sites had more than 1% methylation. In addition, we performed single replica experiments of the same type of high-throughput bisulfite sequencing experiments with the S291F and S317A mutants and observed similar results. The percent trans/cis strand methylation was 21%/2.2% with S291F and 20.6%/3.5% with S317A.

**Fig 9:**
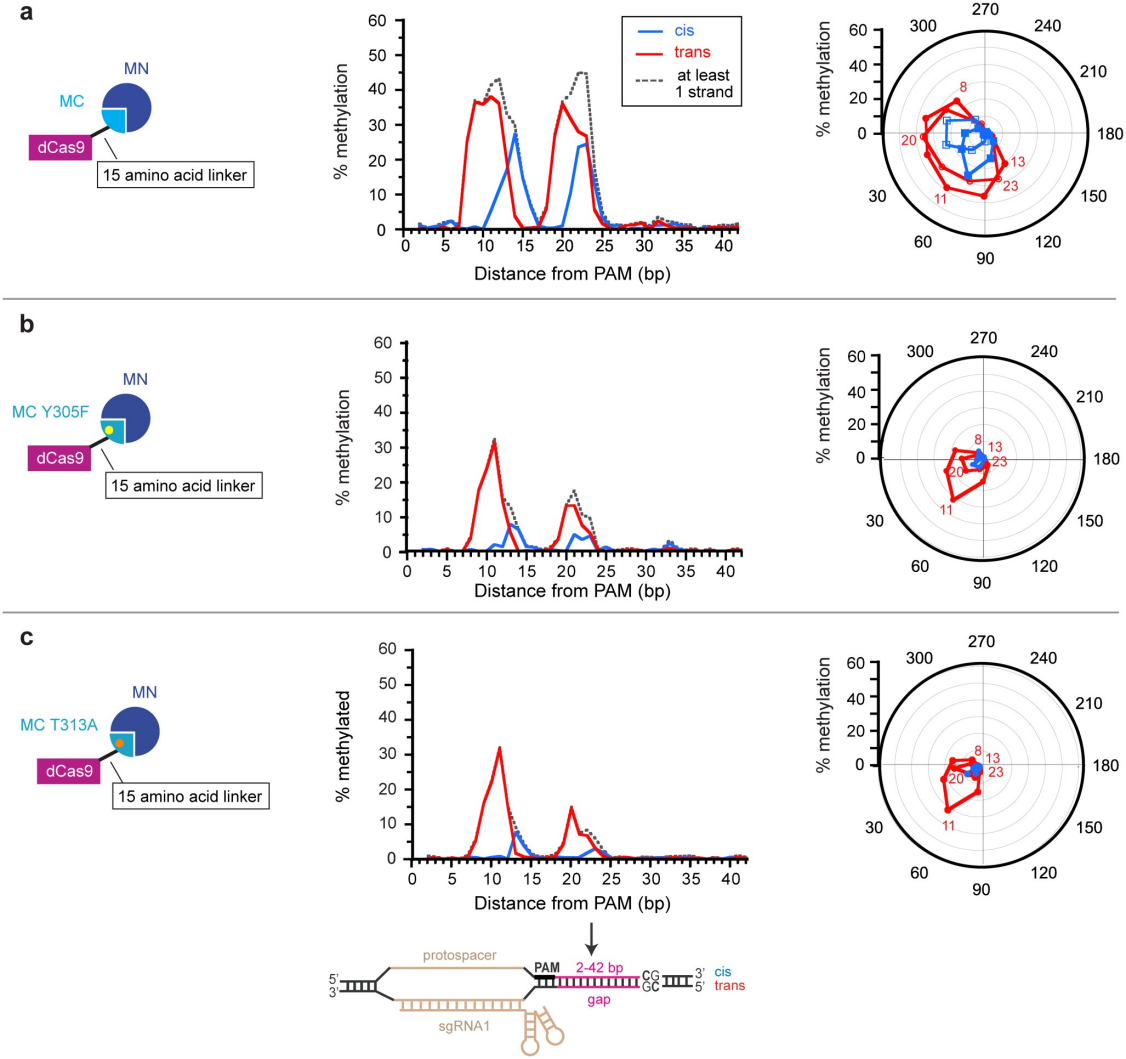
Effect of mutations on methylation in the target region as a function of gap length. Frequency of methylation for cis and trans strand are presented as a function of distance from the PAM (center) and the angle between the dCas9 C-terminus and the MC N-terminus (right) in the structural model for dCas9-MC/MN with a 15-amino acid linker and (a) no mutation, (b) the Y305F mutation in the MC domain, and (c) the T313A mutation in the MC domain. The angle is defined as that formed between the C-terminus of Cas9 and N-terminus of MC when looking down the DNA axis from the CpG site towards the PAM (i.e. the angle formed in the plane of the page in the view of Fig 2b and 2d). The dotted line indicates the fraction of the target sites that are methylated on at least one strand assuming that cis and trans methylation is independent. For b and c, methylation frequency data was not obtained for the trans strand for the gap lengths of 31 and 37 bp. Data in (a) is from Xiong et al. [13].

We next asked how the Y305F and T313A mutations affected methylation of the target site as a function of its distance from the PAM. We compare this data to our previous study [13] with constructs lacking a mutation (Fig 9a). The pattern of methylation for the two mutations was qualitatively similar (Fig 9b and 9c). They restricted the distances between the PAM and the CpG sites that are compatible with methylation (Fig 9b and 9c). The mutations almost eliminated methylation on the cis strand. The highest level of methylation (~30%) occurred on the trans strand at gap of 11 nucleotides, and the methylation level dropped off precipitously at gaps of 10 and 12 nucleotides. As observed when the linker was shortened (Fig 4a), the mutations caused greater decrease in methylation at longer gap distances and at gap distances that are less than optimal for methylation. We reasoned that CpG sites located at non-optimal distances from the PAM site have a lower effective concentration than those at gap distances that are optimal for methylation. Therefore, the mutations’ differential effect at the target region as a function of gap length is consistent with our model of how *K*_m_-increasing mutations can improve the targeting of methylation (Fig 5).

**Fig 10:**
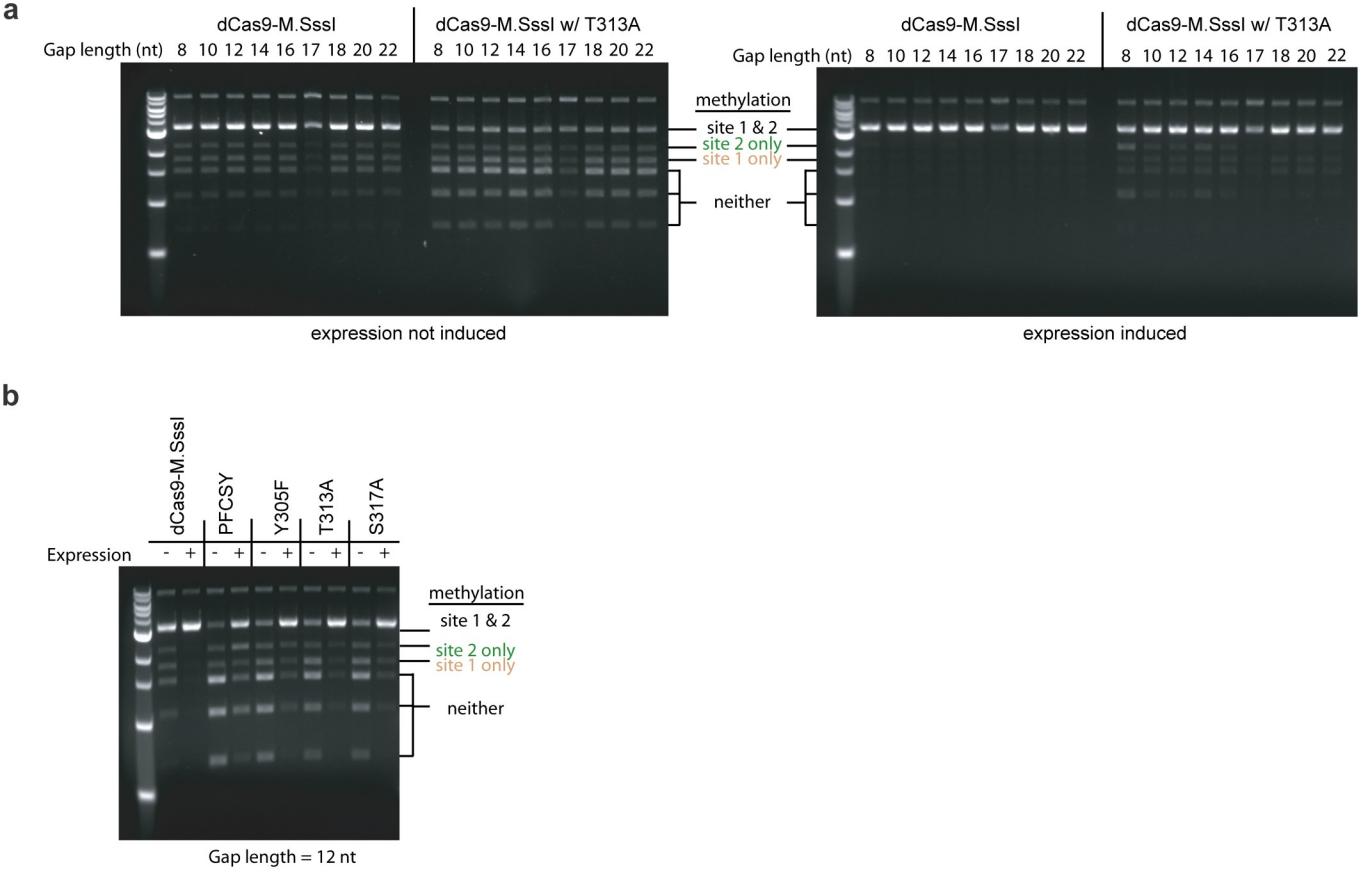
Methylation as caused by dCas9-M.SssI and associated mutants as assessed by the restriction enzyme protection assay. Methylation pattern as a function of mutation and induction of expression (a) as a function of the distance away from the PAM (8–22 bp) with and without the T313A mutation and (b) at a distance of 12 bp away from the PAM for several different mutations. Methylation was assayed from isolated plasmid DNA from ER2267 cells grown in lysogenic broth with 0.2% glucose and either with (+) or without (–) 0.0167% arabinose for induction of expression of dCas9-M.SssI variants under the *pBAD* promoter. Methylation was assayed as described in Fig 1. The size of diagnostic bands for methylation is different than in Fig 1 since MN has been deleted from pReporter (dCas9-M.SssI is expressed from pEncode). sgRNA1 was used to target site 1.

Next, we tested whether the mutations could turn an end-to-end fusion of dCas9 and M.SssI into an effective targeting enzyme for methylation. M.SssI is known to be highly active even when fused to dCas9 [13, 20]. Lei et al characterized the effect of the Q147L mutation in an end-to-end fusion of dCas9 and M.SssI, which they named dCas9-MQ1[20]. This mutation in M.SssI reduces activity through disrupting interactions with DNA [26] and helps methylation targeting of dCas9-MQ1 in mammalian cells[20]. Previously, we found that our end-to-end fusion of dCas9 and M.SssI (dCas9-M.SssI) methylated 100% of both the target and non-target sites in *E*. *coli* [13]. Here, we tested the T313A mutation in dCas9-M.SssI under a variety of induction conditions and gap lengths, and do not find evidence of any methylation targeting (Fig 10a). In all cases, the mutation reduced the level of methylation, but resulted in no discernable targeting of methylation to the target site (Fig 10a). We further tested other mutants in single replica experiments and also observed a reduction in methylation with a lack of targeting (Fig 10b). This suggests that, to a large extent, the M.SssI domain in dCas9-M.SssI is methylating independent of the dCas9 binding, since for an un-tethered M.SssI, we expect that these mutations would decrease methylation efficiency at all CpG sites equally, which is what we observe. We used a longer linker than Lei et al and designed it to be flexible. Our fusion of dCas9 and M.SssI has a 15-amino acid, flexible (GSGGG)_3_ linker, whereas dCas9-MQ1 with the Q147L mutation studied by Lei et al has a linker of about 9 amino acids, most of which also serves as the nuclear localization sequence [20]. They posited that their linker was optimal for reaching 20–30 bp away from the dCas9 binding site. Thus, our fusion cannot be incapable of reaching the target CpG sites we tested due to it being too short or inflexible. Altogether, our findings demonstrate that our mutations improved targeting in the context of the split methyltransferase but not in the context dCas9 fusion protein fused end-to-end with the full-length M.SssI as tested here in *E*. *coli*. The splitting of M.SssI renders the methyltransferase ineffective at methylation, as intended in the design. dCas9 fusion to this otherwise ineffective methyltransferase results in methylation targeted to a desired site, and the addition of the mutations serves to enhance this targeting.

We did not examine how the linker lengths or mutations affected the level of soluble, active dCas9-MC/MN in the cell. Mutations that cause a reduction in the amount of soluble, active enzyme would lower the level of methylation observed, but they cannot explain our results. Changes in the level of the enzyme could not simultaneously increase methylation on the trans strand but decrease methylation on the cis strand, as changing from a 15 aa linker to a 4 aa linker does (Fig 3a), while leaving the levels of off-target methylation relatively unchanged (Fig 3c and 3d). Changes in expression could not change the pattern of methylation seen in Fig 4 or how the mutations preferentially decrease methylation at non-target sites (Fig 8).

## Conclusions

Our findings offer insights and design rules for improving methylation as targeted by dCas9-MC/MN in *E*. *coli*. We demonstrated that shortening the linker length between the dCas9 and the MC fragment increases methylation at the target site positioned at a select range of gap lengths, but resulted in little to no reduction in non-target site methylation except in the immediate vicinity of the target site. To preferentially reduce methylation at more distant non-target sites, we used mutations designed to reduce the catalytic activity of the methyltransferase domain by reducing DNA affinity. These mutations served to move the enzyme into a regime where, in the context of the cell, the preferential methylation of target site over non-target site can be best achieved.

Highly-selective methyltransferases will enable the further understanding of CpG methylation and its cellular role. We have previously shown how our methylation targeting design rules for dCas9-MC/MN in *E*. *coli* are generalizable to mammalian systems. Based on these previous findings, we hypothesize that the linkers/mutations we identify here will be beneficial in mammalian cells; however, testing in mammalian cells will be required to evaluate this hypothesis. If our hypothesis is confirmed, we anticipate that our mutant dCas9-MC/MN will be especially useful in mammalian systems when very precise methylation is needed. For example, interrogation of methylation’s connection with exon usage would benefit from such precise control of methylation [16, 30]. In addition, precise targeting of methylation might be useful to alter the expression of genes that are regulated by transcription factors whose affinity for DNA is modulated by cytosine methylation [31]. Applying these linkers/mutations in mammalian systems might be particularly important to reduce methylation at unintended sites and could augment efforts to reduce undesired methylation due to off-target dCas9 binding. The linkers/mutations might be less useful at loci where complete methylation of an entire CpG island is necessary to effect changes in expression.

## Materials and methods

### Design of dCas9-sMTase proteins with different linker lengths

The pEncode plasmid containing dCas9-MC gene and sgRNA, along with its J23100 promoter and terminators [13] served as the template for the construction of new dCas9-MC genes. The insertion of different linkers between dCas9 and MC was created by designing primers with sequence identity to the linkers on the 5’ end, to amplify and linearize the plasmid using inverse PCR. Primers were obtained from IDT and phosphorylated with T4 Polynucleotide Kinase (New England Biolabs, Ipswich, MA, USA). PCR amplification was done using Phusion Master Mix (New England Biolabs, Ipswich, MA, USA), and the PCR product containing the linearized plasmid was purified using PureLink Gel Extraction Kit (Thermo Fisher Scientific, Waltham, MA, USA). The linearized plasmid was ligated using either T4 Ligation Kit (New England Biolabs, Ipswich, MA, USA), or Gibson Assembly [32] prepared using Taq Ligase from New England Biolabs (NEB). Ligation products were then used to transform chemically cells made from competent *E*. *coli* K12 ER2267 cells (F´ *proA*^+^*B*^+^ *lacI*^*q*^ *Δ(lacZ)M15 zzf*::*mini-Tn*10 (Kan^R^)/ *Δ(argF-lacZ)U169 glnV44* e14^-^(McrA^-^) *rfbD1*? *recA1 relA1*? *endA1 spoT1*? *thi-1 Δ(mcrC-mrr)114*::*IS10)* from NEB. Plasmid DNA was isolated from the colonies recovered from the transformation plate containing 50 μg/mL of chloramphenicol (Sigma, St. Louis, MO, USA). Co-transformed ER2267 cells with this plasmid and either the pReporter or the gap library, as previously described [13], were plated on plates containing 1% w/v glucose, 100 μg/mL ampicillin, and 50 μg/mL chloramphenicol.

### Design of dCas9-sMTase and dCas9-MTase proteins with single amino acid substitution

The pEncode plasmid containing dCas9-MTase gene or the dCas9-MC served as the template for the construction of genes encoding dCas9-MTase mutant or dCas9-MC, respectively. Site mutagenesis in the dCas9-MC gene was created by designing primers containing the mutations in the 5’ end that were used to amplify and linearize the plasmid using inverse PCR. The linearized plasmid was purified as described above, and then phosphorylated and ligated with T4 Polynucleotide Kinase and T4 Ligation Kit, respectively. Ligation products were transformed into chemically competent ER2267 cells as aforementioned.

### Restriction enzyme protection assay

A 5–10 mL lysogenic broth containing standard supplements (0.2% w/v glucose, 100 μg/mL ampicillin, and 50 μg/mL chloramphenicol), 0.0167% w/v arabinose, and 1 mM IPTG was inoculated from 1 μL of glycerol stock. Arabinose induced expression of dCas9-MC, and IPTG induced expression of MN. The culture was shaken at 250 rpm at 37°C for 16–18 hours. Cultures were centrifuged at 2880 x g for 7 minutes, and plasmids were extracted from pelleted cells using the Plasmid Miniprep Kit (Qiagen, Germantown, MD, USA). Plasmid DNA (180 ng) was incubated with 10 units FspI and 20 units SacI-HF in 10 μl Cutsmart Buffer (NEB) at 37°C for 1.5 hours. SacI-HF was used for plasmid linearization. The digested DNA was loaded into a 1.2% w/v TAE gel containing ethidium bromide, and electrophoresed at 110 Volts for 50 minutes. Ultrapure agarose was obtained from Thermofisher Scientific. Band patterns were visualized under UV light and imaged with Carestream Gel Logic 112.

### Analysis of methylation using high-throughput bisulfite sequencing

Glycerol stocks (1 μL) were used to inoculate 5 mL lysogenic broth containing the standard supplements. Cultures were shaken at 250 rpm at 37 °C for 15 hours. From the overnight culture, 1 mL was transferred into a 500 mL shake flask containing 150 mL lysogenic broth with the standard supplements and shaken at 250 rpm at 37°C. The cultures were supplemented with 0.0175% w/v arabinose and 1mM IPTG at an optical density of 0.3, and shaken for 4 hours at 250 rpm at 37°C. A 5 mL aliquot of the culture was centrifuged, and plasmid DNA was extracted from the pelleted cells using the Plasmid Miniprep Kit. Preparation of the purified DNA for high-throughput bisulfite sequencing was as described [13]. Briefly, DNA was sheared to 300 bp (Diagenode Bioruptor Pico), then end repaired and methylated adaptors ligated using NEBNext Ultra (NEB). Bisulfite treatment was performed with EZ Methylation Lightning (Zymo) then amplified with Kapa Hifi Uracil+ ReadyMix from Kapa Biosystems and NEBNext Multiplex Oligos for Illumina (Methylated Adaptor) from NEB for 8 cycles. DNA concentration was determined using qPCR (Kapa Illumina Library Quantification Kit), and size distribution was confirmed using the High Sensitivity Bioanalyzer. Libraries were subsequently sequenced on an Illumina MiSeq using v3 chemistry. Data was deposited in SRA under Bioproject PRJNA503938.

### Analysis of MiSeq data

MiSeq bisulfite sequencing data was aligned to a reference sequence for the plasmid using bowtie2 via Bismark [33]. After alignment, methylation data averaged across all reads and strand and context specific methylation information was extracted with custom R scripts (https://github.com/timp0/xiong_splitcas9).
